# Primary uterine ectomesenchymoma harboring a *DICER1* mutation: case report with molecular analysis

**DOI:** 10.1007/s00428-021-03057-x

**Published:** 2021-02-17

**Authors:** Ben Davidson, Lilach Kleinberg, Ida Marie Børresen, Frøydis Slettevoll, Anne Fangberget, Dunia Hindosh, Ane Gerda Zahl Eriksson, Bodil Bjerkehagen

**Affiliations:** 1 Department of Pathology, Oslo University Hospital, Norwegian Radium Hospital, Montebello, N-0310 Oslo, Norway; 2 Faculty of Medicine, Institute of Clinical Medicine, University of Oslo, N-0316 Oslo, Norway; 3 Department of Radiology, Oslo University Hospital, Norwegian Radium Hospital, N-0310 Oslo, Norway; 4 Department of Radiology, Kalnes Hospital, Kalnesveien 300, 1714 Grålum, Norway; 5 Department of Gynecologic Oncology, Oslo University Hospital, Norwegian Radium Hospital, N-0310 Oslo, Norway; 6 Institute of Oral Biology, University of Oslo, N-0310 Oslo, Norway

**Keywords:** Ectomesenchymoma, Uterus, Immunohistochemistry, Massive parallel sequencing, Metastasis

## Abstract

Ectomesenchymoma is an exceedingly rare biphasic malignant tumor characterized by the presence of mesenchymal and neuroectodermal elements. The majority of patients are infants or children. We describe the first case of this entity diagnosed as a primary uterine tumor. A 72-year-old female presented with post-menopausal bleeding. Dilatation and curettage showed irregular mesenchymal proliferation of uncertain nature. In the hysterectomy specimen, a myxoid spindle cell tumor with areas of skeletal muscle and neural differentiation was found in the uterus, with direct invasion of the small intestine, and biphasic differentiation into rhabdomyosarcoma and ganglioneuroblastoma was unequivocally seen in a lymph node metastasis. The morphological findings were validated by immunohistochemistry. Massive parallel sequencing identified *TP53*, *PTEN*, and *DICER1* mutations in the tumor. This report describes the presence of ectomesenchymoma in an unusual primary organ and in an uncharacteristic age and presents novel data regarding the genetic characteristics of this tumor.

**Supplementary Information:**

The online version contains supplementary material available at 10.1007/s00428-021-03057-x.

## Introduction

Ectomesenchymoma is an exceedingly rare biphasic malignant tumor characterized by the presence of mesenchymal and neuroectodermal elements. The former manifests as rhabdomyosarcoma (RMS), which may have botryoid, spindle cell and myxoid, or primitive round cell pattern. The neuroectodermal component is neuroblastic, with variable degree of differentiation, manifesting as neuroblastoma, ganglioneuroblastoma, or ganglioneuroma. The majority of patients are infants or children younger than 15 years, often <5 years, with a male-to-female ratio of 1.38. Common primary sites include the pelvis/perineum, urogenital organs, and intra-abdominal or retroperitoneal soft tissue. A minority of tumors is located at the head and neck region, the extremities, and the mediastinum. Tumors have been reported to have a diameter of 3–18 cm, with tan color and frequent hemorrhage and necrosis. Treatment and outcomes are comparable to those of patients with embryonal RMS. Parameters associated with favorable outcome include age ≤3 years, size <10 cm, and superficial location [1].

To the best of our knowledge, ectomesenchymoma has not been reported as a primary uterine tumor. We report a rare case of this malignancy presenting at this anatomic location, at an unusual age. We additionally report on the genomic profile of this tumor.

## Case report

### Clinical presentation

A 72-year-old female presented to her local gynecologist with post-menopausal bleeding. A uterine tumor was found, clinically diagnosed as leiomyoma. Her past medical history included insulin-dependent diabetes mellitus, hypertension, and hypercholesterolemia, with no previous gynecologic history and 6 term deliveries. Following inconclusive diagnosis after dilatation and curettage (see below), the patient was referred to the Department of Gynecologic Oncology at the Norwegian Radium Hospital. At presentation, the patient reported continuous pinkish vaginal discharge and loss of energy and possibly weight over the last few weeks, with no abdominal discomfort. Her physical examination was unremarkable, performance status ECOG 0-1. Pelvic examination revealed a large, non-painful immobile pelvic/abdominal mass. CA125 was within normal range (24U/mL), and HE4 was elevated (188 pmol/L).

**Fig. 1:**
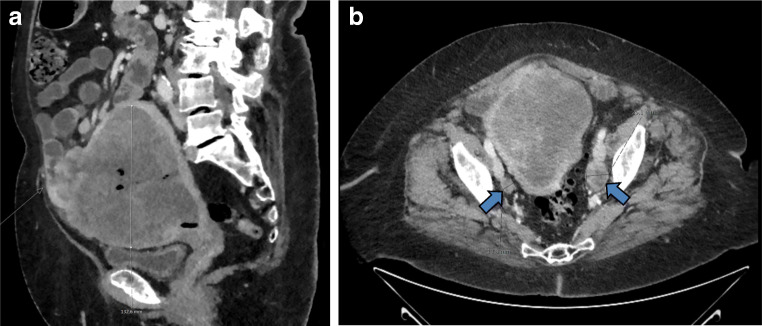
Radiology. **a** Sagittal CT showing a large heterogeneous tumor filling the uterine cavity, with ventral invasion of the uterine wall and adjacent small intestine loop. Black areas in the tumor represent air, result of biopsy or intestinal perforation, **b** Axial CT of the pelvis showing enlarged lymph nodes (arrows)

Chest/abdomen/pelvic CT and pelvic MRI demonstrated significantly enlarged uterus with a large (11.6 × 11.9 × 13.4 cm), heterogeneous tumor and enlarged iliac lymph nodes. The uterine tumor infiltrated the myometrium and breached the serosa, with suspected invasion of adjacent small bowel. CT demonstrated an air-filled area thought to represent fistula to the adjacent small bowel (Fig. 1). CT also showed pulmonary nodules up to 6 mm in diameter suspicious for metastasis.

The patient was assessed to be surgical candidate with good performance status, and complete resection of the abdominal disease was deemed feasible. Surgery consisted of en bloc resection of the uterus, adnexae, and two small bowel loops adherent to the uterus, as well as removal of enlarged pelvic lymph nodes. At the end of surgery, there was no gross residual disease intra-abdominally, with clear surgical margins achieved and no tumor spillage.

### Histopathology

**Fig. 2:**
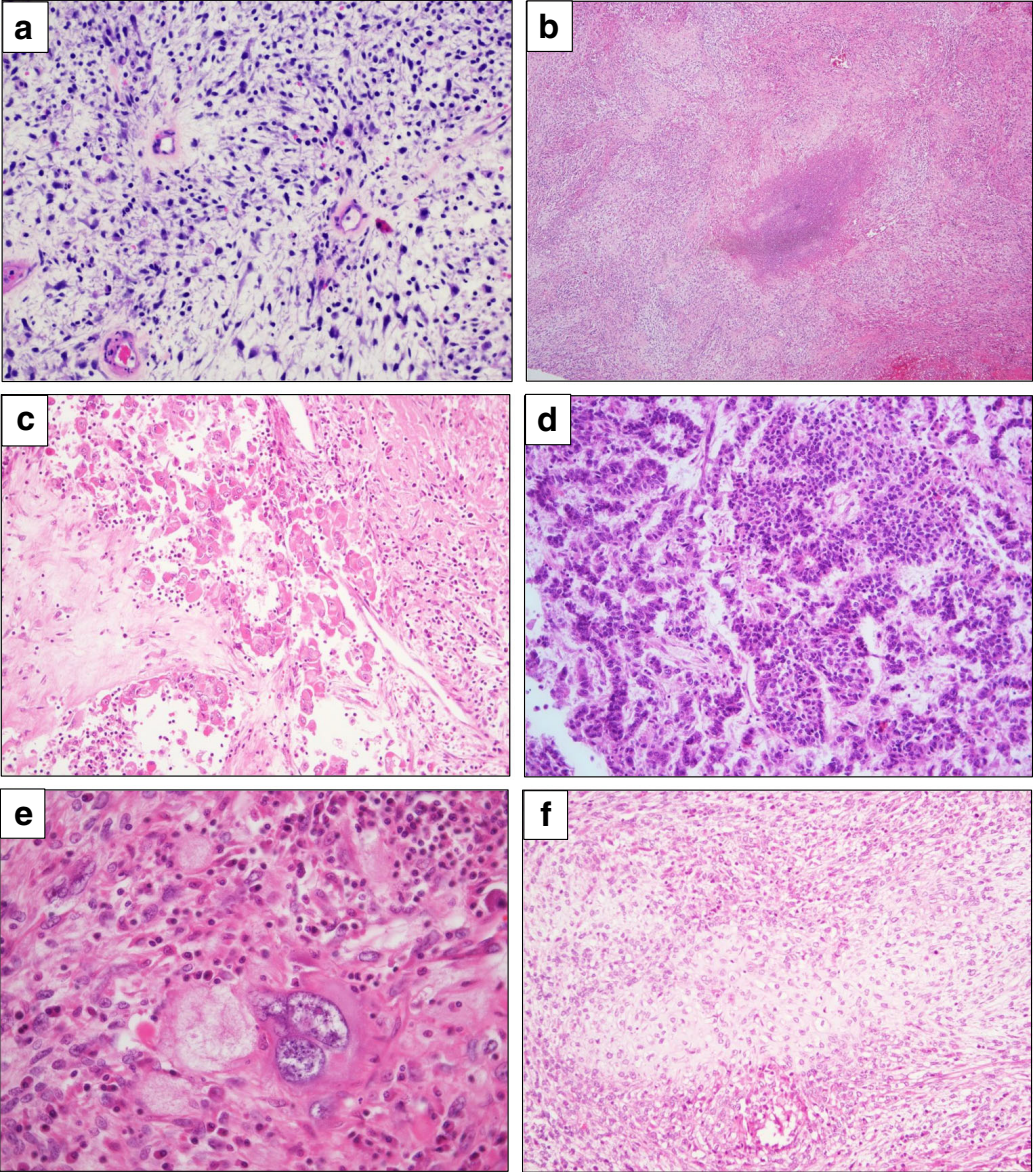
Uterine tumor. **a** Myxoid irregular spindle cell proliferation in the curettage specimen, **b** Myxoid areas and foci with necrosis in the hysterectomy specimen, **c** Skeletal muscle differentiation. **d** Epithelioid acini, trabeculae, and rosette-like structures, **e** Severe atypia, **f** Cartilage formation

In the preoperative curettage specimen, a partly myxoid irregular spindle cell proliferation, with areas of necrosis and pronounced inflammation, was observed (Fig. 2a). Immunostaining showed expression of vimentin and CD10, negative staining for pan-cytokeratin AE1/AE3, desmin, caldesmon, SMA, actin, ALK, and p16. p53 showed wild-type pattern (data not shown). No conclusive diagnosis of malignancy or typing of the process was deemed possible.

Gross examination of the surgical specimen showed a uterine tumor measuring 12.5 cm in largest diameter, with areas of hemorrhage and necrosis. Breach of the uterine serosa and invasion of adjacent small intestine was evident. Microscopically, a spindle cell proliferation reminiscent of the curettage specimen was seen, with myxoid areas and foci of necrosis (Fig. 2b). Cells with abundant eosinophilic cytoplasm suggestive of the skeletal muscle differentiation (Fig. 2c) and epithelioid acini, trabeculae, and rosette-like structures (Fig. 2d) were additionally seen, as were areas of increased, focally severe atypia (Fig. 2e) and evidence of cartilage formation (Fig. 2f). Mitotic counts were 8/10 HPF, some atypical.

**Fig. 3:**
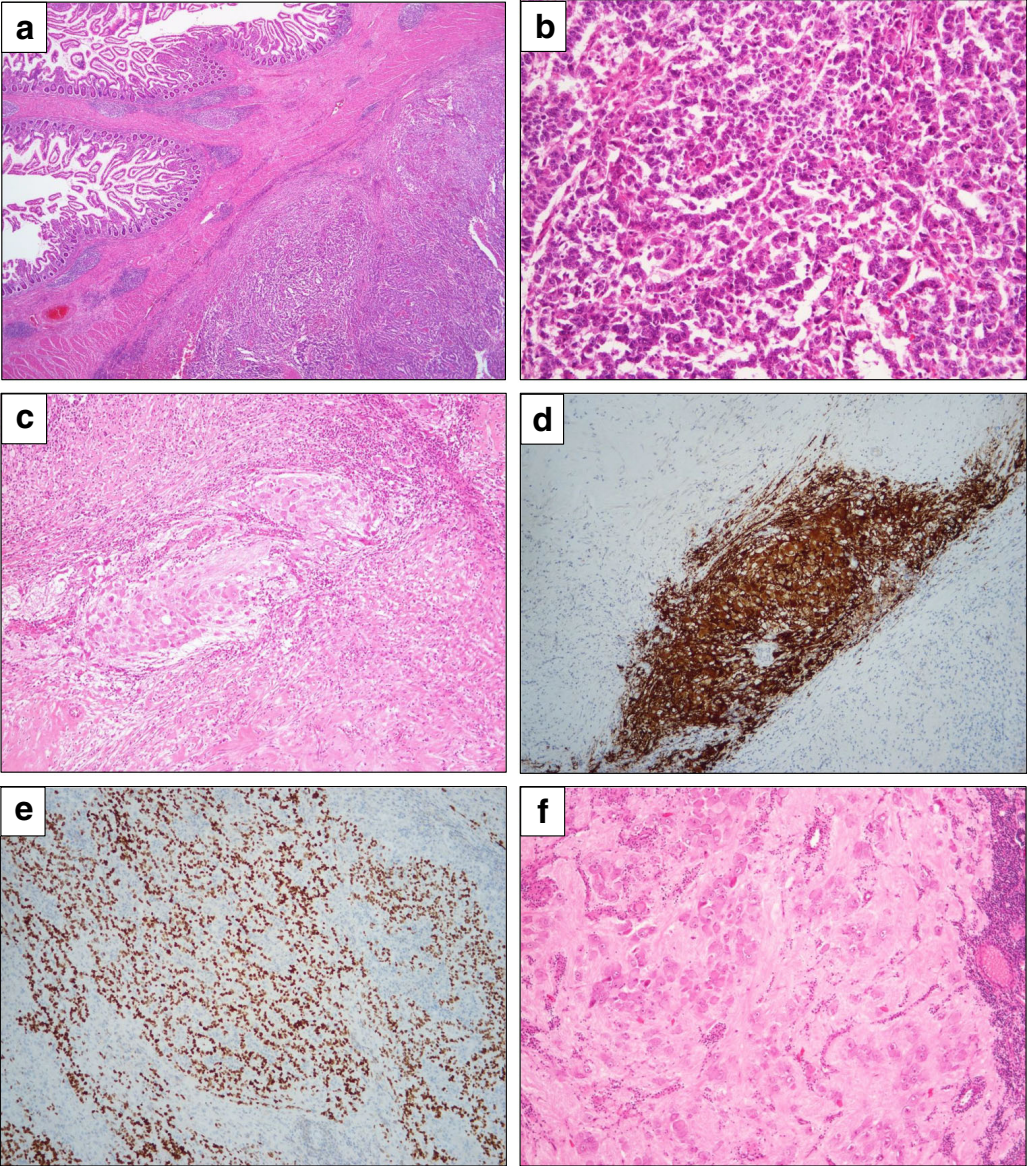

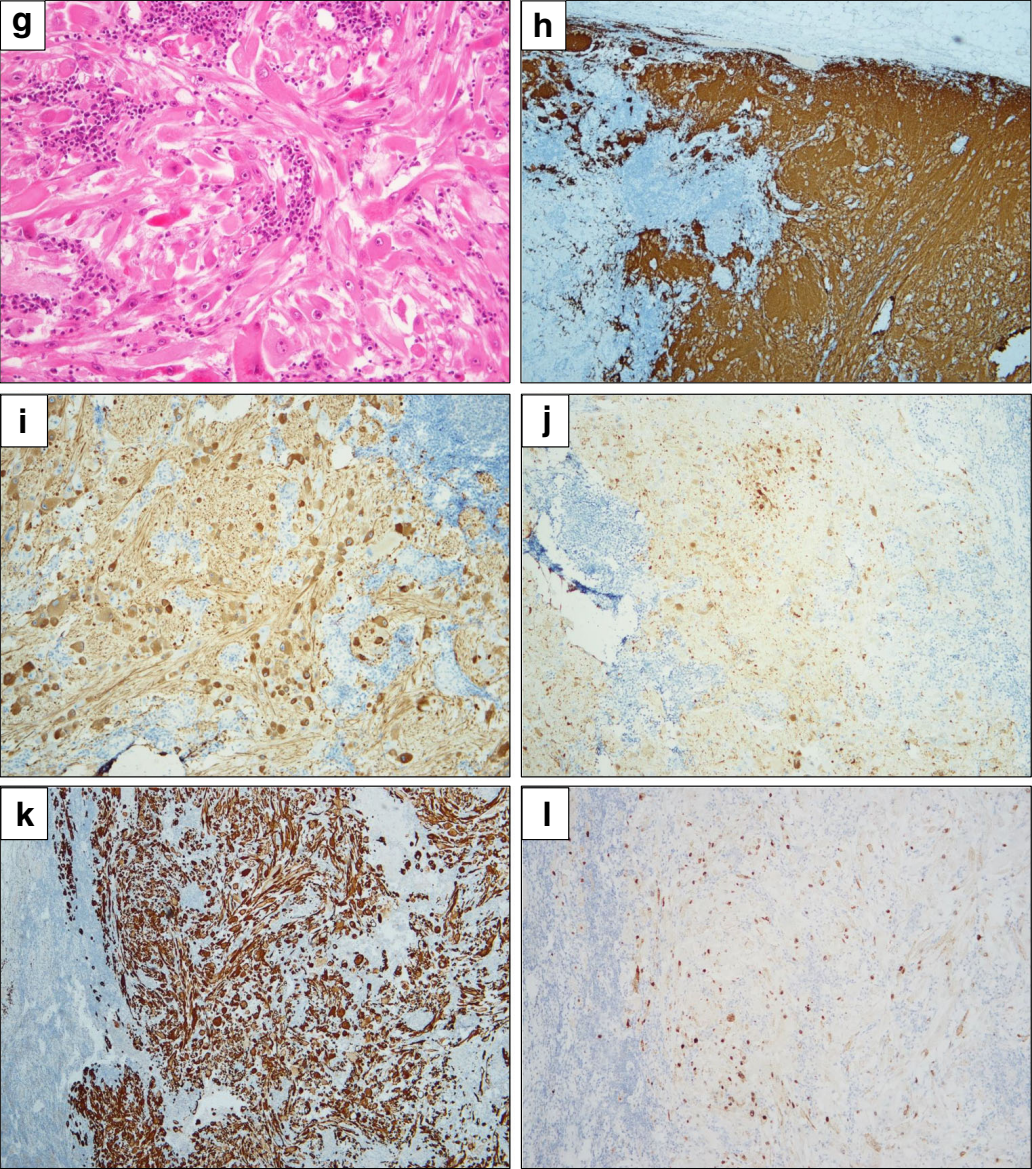
Small intestine invasion and lymph node metastasis. Small intestine. **a** tumor invasion of the small intestine, **b** epithelioid acini, trabeculae, and rosette-like structures, suggesting neural differentiation, **c** mature ganglion cells, **d** positive immunostaining for synaptophysin; and **e** positive immunostaining for SALL4. Left obturator lymph node, **f** ganglion cells, **g** skeletal muscle, **h–j** positive immunostaining for synaptophysin (**h**), NF (**i**), and CGA (**j**) in the neural cells, **k-l** positive immunostaining for desmin (**k**) and Myf-4 (**l**) in the skeletal muscle cells

As assessed clinically, radiologically and at gross examination, the tumor invaded the small intestine (Fig. 3a), where the predominant histology was of the abovementioned epithelioid acini, trabeculae, and rosette-like structures (Fig. 3b), as well as clusters of mature ganglion cells (Fig. 3c). Neural, predominantly immature differentiation was confirmed by positive immunostaining for synaptophysin (Fig. 3d) and SALL4 (Fig. 3e), respectively, with only focally positive (<5%) CK8 expression and negative staining for Ber-EP4. Staining for chromogranin A (CGA) and OCT3/4 was negative.

Metastases were found in 2/6 removed lymph nodes, one in the left obturator region and one in the right external iliac region. In the former, unequivocal differentiation into ganglion cells (Fig. 3f) and skeletal muscle (Fig. 3g) was found, confirmed by positive immunostaining for synaptophysin (Fig. 3h), pan-neurofilament (NF; Fig. 3i), and CGA (Fig. 3j) in the neural cells; desmin (Fig. 3k) and Myf-4 (Fig. 3l) in the skeletal muscle cells. Both components were positive for vimentin. S100 staining was focally positive.

### Massive parallel sequencing

DNA and RNA were extracted from sections of formalin-fixed, paraffin-embedded tissue (FFPE) using QIAamp DNA FFPE Tissue Kit (Qiagen, Hilden, Germany), and RNeasy FFPE Kit (Qiagen), respectively. Tumor cell percentage in the sections was estimated at 90%. Targeted sequencing of 203 cancer-associated genes was performed using the Oncomine Childhood Cancer Research Assay on an Ion Torrent S5 Prime instrument (both from Thermo Fisher Scientific, Carlsbad, CA). Sequences were analyzed for mutations, copy number gain, and gene fusions using Ion Reporter Software v.5.10 (Thermo Fisher Scientific). DNA was additionally analyzed for *TERT* promoter mutations using Sanger sequencing.

Variant analysis revealed c.5428G>T; p.Asp1810Tyr *DICER1* missense mutation, missense mutation in *TP53* (c.730G>A; p.Gly244Ser), and two missense mutations in *PTEN* (c.376G>A; p.Ala126Thr and c.406T>C; p.Cys136Arg). No fusion genes, copy number gain, or *TERT* promoter mutations were detected. Microdissection of the RMS and neuroectodermal component, with repeat of the analysis, showed that all four mutations were more frequent in the neuroectodermal component (Supplementary Table 1).

### Follow-up

CT of the chest/abdomen/pelvis 6 weeks postoperatively demonstrated pulmonary embolus, left-sided pleural effusion, and slight regression of the previously detected pulmonary nodules. New peritoneal thickening consistent with sarcomatosis was found. The patient was offered palliative chemotherapy. The patient has recently completed her second cycle of adjuvant chemotherapy, consisting of Doxorubicin (50 mg/m^2^) and Ifosfamide (5 g/m^2^). A decision has been made to continue with Doxorubicin monotherapy for the remaining cycles due to adverse events related to Ifosfamide. CT has been scheduled after her third cycle to assess chemotherapy response.

## Discussion

The majority of patients diagnosed with ectomesenchymoma are pediatric [2–5]. Though isolated reports of tumors diagnosed in adults have been published [6, 7], the age of the patient in this report is exceptional. Similarly, whereas location of the primary tumor in the abdomen, pelvis, groin, perineum, paratesticular region, and vulva has been reported [2–5], ectomesenchymoma has not, to the best of our knowledge, been diagnosed as a primary uterine tumor to date. One possibility which cannot be entirely ruled out is that this tumor developed from a carcinocarcinoma. However, no epithelial component was found in this tumor despite extensive sampling.

Previous molecular studies of ectomesenchymoma have shown chromosomal changes (trisomy, polyploidy, or focal gains) involving chromosomes 2, 6, 8, 9, 10, 11, and 20, as well as t(12;15)(p12.3;q24.1) and t(1;12)(p32;p13) fusions, the latter with no rearrangement of *ETV6* [8–12]. Griffin et al. found *FOXO1* gene rearrangements by RT-PCR in three cases with alveolar RMS morphology, of which two involved *PAX3-FOXO1* fusion, and one harbored *PAX7-FOXO1* translocation [5]. Whole genome/exome sequencing studies of this tumor are to date limited to one report. Huang and co-workers studied seven ectomesenchymomas by RNA sequencing and detected *HRAS*, *PTPRD*, and *FBXW7* mutations in six, two, and one cases, respectively. No fusion genes were found [4].

In the present study, we performed sequencing analysis of FFPE material from the patient’s lymph node metastasis, which contained tumor with both skeletal muscle and neural differentiation. We report the presence of missense mutations in *DICER1*, *TP53*, and *PTEN*, the latter with two different mutations. Three of the four mutations have been reported to be pathogenic in various cancers (*TP53*: https://www.ncbi.nlm.nih.gov/clinvar/variation/VCV000376600.5; *PTEN*: https://www.ncbi.nlm.nih.gov/clinvar/variation/VCV000183726.6; *DICER1*: https://www.ncbi.nlm.nih.gov/clinvar/variation/VCV000933084.1), whereas the second PTEN mutation is of uncertain pathogenicity (https://www.ncbi.nlm.nih.gov/clinvar/variation/VCV000468683.4) (Database accessed Dec. 1 and Dec. 6, 2020).

Whether these mutations are germline or somatic cannot be assessed with certainty, as peripheral blood was not available for mutation analysis. However, the patient’s age at disease presentation and variant allele frequency <20% for all detected mutations in whole tissue section analysis are suggestive of somatic origin. The *DICER1* mutation, previously not described in ectomesenchymoma, may be of particular interest, as mutations in this gene have been described in embryonal RMS, as well as in RMS, not otherwise specified (NOS), anaplastic sarcoma, and sarcoma NOS [13]. The specific mutation found in the present tumor, c.5428G>T; p.Asp1810Tyr, has been reported in the uterine and cervical embryonal RMS and in uterine and peritoneal adenosarcoma, as well as in two embryonal RMS of unknown origin [13], reinforcing the link between ectomesenchymoma and embryonal RMS highlighted in the Huang paper [4]. Whether any of these tumors was an ectomesenchymoma in which the neural component was not sampled remains an open question.

In conclusion, the present study reports a primary ectomesenchymoma of the uterus, presenting at an uncharacteristic age and anatomic site, with mutations in *DICER1*, *TP53*, and *PTEN*. This malignancy, as RMS, is generally associated with unfavorable outcome when diagnosed with distant spread, and its management may benefit from future molecular characterization.

## Supplementary Information

ESM 1 (DOCX 22 kb)

## Declarations

The patient signed an informed consent form approving the use of tissue for the present study and publication of the data, according to the guidelines of Oslo University Hospital.
