# Supplementary material for: Primary uterine ectomesenchymoma harboring a DICER1 mutation: case report with molecular analysis
Source: Virchows Arch. 2021 Feb 17;479(2):419–24. doi: 10.1007/s00428-021-03057-x (PMC8364549; doi:10.1007/s00428-021-03057-x)
Supplement: Supplementary file 1 — (DOCX 22 kb) [file 428_2021_3057_MOESM1_ESM.docx]

**Supplementary Table 1: Mutation frequency in the different tumor components**

| **Gene** | **Variant** | **Frequency (%) in whole section** | **Frequency (%) in N-component** | **Frequency (%) in R-component** |
| --- | --- | --- | --- | --- |
| *PTEN* | c.376G>A; p.Ala126Thr | 14.6 | 68.0 | 5.1 |
| *PTEN* | c.406T>C; p.Cys136Arg | 6.9 | 20.9 | ND |
| *DICER1* | c.5428G>T; p.Asp1810Tyr | 18.2 | 54.5 | 10.2 |
| *TP53* | c.730G>A; p.Gly244Ser | 6.9 | 45.0 | 5.9 |

**Abbreviations:** N-component **=** neuroectodermal component; R-component **=** rhabdosarcomatous component; ND **=** Not detected
